# Reactivity of redox sensitive paramagnetic nitroxyl contrast agents with reactive oxygen species

**DOI:** 10.3164/jcbn.17-135

**Published:** 2018-09-15

**Authors:** Minako Nyui, Ikuo Nakanishi, Kazunori Anzai, Toshihiko Ozawa, Ken-ichiro Matsumoto

**Affiliations:** 1 Quantitative RedOx Sensing Team, Department of Basic Medical Sciences for Radiation Damages, National Institute of Radiological Sciences, National Institutes for Quantum and Radiological Science and Technology, 4-9-1 Anagawa, Inage-ku, Chiba 263-8555, Japan; 2 Division of Physical and Analytical Chemistry, Nihon Pharmaceutical University, 10281 Komuro, Ina-machi, Kitaadachi-gun, Saitama 362-0806, Japan; 3 Laboratory of Oxidative Stress Research, Showa Pharmaceutical University, 3-3165 Higashi-tamagawagakuen, Machida, Tokyo 194-8543, Japan

**Keywords:** nitroxyl radical, superoxide, hydroxyl radical, hydroperoxyl radical, one-electron oxidation

## Abstract

The reactivity of nitroxyl free radicals, 4-hydroxyl-2,2,6,6-tetramethylpiperidine-*N*-oxyl (TEMPOL) and 3-carbamoyl-2,2,5,5-tetramethylpyrrolidine-*N*-oxyl (CmP), with reactive oxygen species (ROS) were compared as typical 6-membered and 5-membered ring nitroxyl compounds, respectively. The reactivity of the hydroxylamine forms of both these nitroxyl radicals (TEMPOL-H and CmP-H) was also assessed. Two free radical species of ROS, hydroxyl radical (^•^OH) and superoxide (O_2_^•−^), were subjected to a competing reaction. ^•^OH was generated by UV irradiation from an aqueous H_2_O_2_ solution (H_2_O_2_-UV system), and O_2_^•−^ was generated by a reaction between hypoxanthine and xanthine oxidase (HX-XO system). ^•^OH and O_2_^•−^ generated by the H_2_O_2_-UV and HX-XO systems, respectively, were measured by electron paramagnetic resonance (EPR) spin-trapping, and the amount of spin adducts generated by each system was adjusted to be equal. The time courses of the one-electron oxidation of TEMPOL, CmP, TEMPOL-H, and CmP-H in each ROS generation system were compared. A greater amount of TEMPOL was oxidized in the HX-XO system compared with the H_2_O_2_-UV system, whereas the reverse was observed for CmP. Although the hydroxylamine forms of the tested nitroxyl radicals were oxidized evenly in the H_2_O_2_-UV and HX-XO systems, the amount of oxidized CmP-H was approximately 3 times greater compared with TEMPOL-H.

## Introduction

**Fig. 1:**
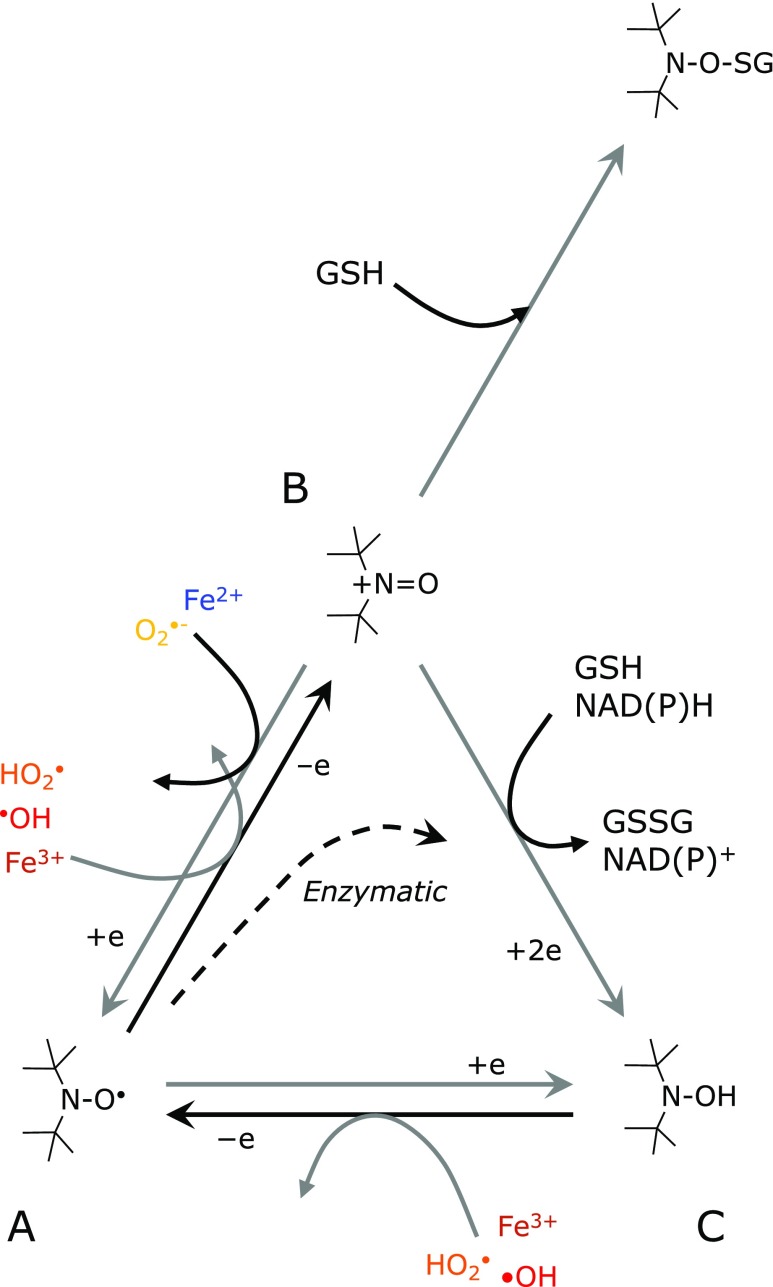
Redox transformations of nitroxyl radicals in (A) the free radical state, (B) the oxoammonium cation, and (C) the hydroxylamine.

Nitroxyl radicals can undergo redox transformations and form hydroxylamine via oxoammonium cations or one-electron reductions (Fig. 1). The paramagnetic nitroxyl radical can be converted to form diamagnetic hydroxylamine by reduction or via the oxoammonium cation *in vivo*. Relatively strong reductants, such as ascorbic acid, can directly reduce nitroxyl radicals by one electron reductions to form hydroxylamine. The nitroxyl radical is commonly oxidized by oxidants, such as a hydroxyl radical (^•^OH), hydroperoxyl radical (HO_2_^•^), and/or Fe^3+^ ion, to form the oxoammonium cation. The oxoammonium cation can then sequentially receive a hydrogen atom from a coexisting hydrogen-donor (H-donor), such as reduced glutathione (GSH), reduced β-nicotinamide adenine dinucleotide (NADH), or reduced β-nicotinamide adenine dinucleotide phosphate (NADPH) to form hydroxylamine. This two-step reduction of nitroxyl radicals to hydroxyl amine, i.e., one-electron oxidation followed by two-electron reduction, can be processed enzymatically *in vivo*. Hydroxylamines can be slowly oxidized by oxidants, such as ^•^OH, HO_2_^•^, and/or Fe^3+^ ions, to re-form nitroxyl radicals. In addition, GSH may also directly and irreversibly react with oxoammonium cations to form a stable complex.^(1)^

Superoxide dismutase (SOD) mimetic reactions have been reported for 6-membered ring-type nitroxyl radicals.^(2,3)^ The redox pair of nitroxyl radicals and oxoammonium cation states underlie its SOD mimic effects.^(4)^ The nitroxyl radical can be one-electron-oxidized by HO_2_^•^, which exists in equilibrium with superoxide (O_2_^•−^) in an aqueous environment. Furthermore, the oxoammonium cation can be one-electron-reduced by O_2_^•−^. This cycling redox reaction is able to function as a SOD mimic.

A common nitroxyl radical, 4-hydroxy-2,2,6,6-tetramethylpiperidine-*N*-oxyl (TEMPOL), has gained attention as a new class of normal tissue-selective radio-protector.^(3,5–8)^ This tissue selectivity is closely associated with the *in vivo* redox mechanisms of nitroxyl radicals. Although nitroxyl radicals may have direct radioprotective effects, hydroxylamines do not.^(3,5)^ The addition of a free radical form of TEMPOL to cell culture showed radioprotective effects in a dose-dependent manner.^(3)^ In contrast, the addition of hydroxylamine in the form of TEMPOL (TEMPOL-H) did not show any radioprotective effects at 100 mM.^(3)^ However, the administration of TEMPOL-H in mice showed similar radioprotective effects to TEMPOL,^(5)^ because TEMPOL-H is re-oxidized to form TEMPOL *in vivo*. The concentration of the free radical forms in the blood increased even after the administration of hydroxylamine.^(5)^ The pharmacokinetic curves of the free radical form in blood, after the administration of TEMPOL-H and that of TEMPOL, almost overlapped, 15 min after the administration and later.^(5)^

TEMPOL has been shown to protect C3H mice against whole-body radiation-induced bone marrow failure.^(6)^ Tumor growth curves generated after 10 and 33.3 Gy of radiation showed no difference in growth between TEMPOL- and PBS-treated animals.^(7)^ In another study, TEMPOL was evaluated for potential differences in radiation protection in salivary glands and in tumors using fractionated radiation.^(8)^ Five daily 6 Gy fractions to the head region of mice reduced saliva production by 54%; however, these effects were significantly reduced by 5 daily treatments with TEMPOL. These results suggested that TEMPOL may protect the salivary tissues from radiation. Because TEMPOL treatment had no effect on radiation-induced tumor regrowth, TEMPOL may work as a selective radioprotector for normal tissue. The difference in radioprotection between normal and tumor tissues may be due to TEMPOL being reduced or cleared from the tumor tissue faster than in normal tissues.

Another common nitroxyl radical, 3-carbamoyl-2,2,5,5-tetramethylpyrrolidine-*N*-oxyl (CmP), has been used as a redox sensitive molecular probe and contrast agent.^(9–12)^ It is also an *in vivo* radioprotector against whole-body radiation.^(13,14)^ Evidence for fast decay rates of CmP in tumor tissues have been obtained by electron paramagnetic resonance (EPR) spectroscopic and imaging experiments. CmP decayed faster in RIF-1 and SCC tumors compared with normal muscle tissues.^(15–17)^ The moderate *in vivo* decay rate of CmP was suitable for EPR experiments.

Paramagnetic nitroxyl radicals have a proton T_1_-shortening effect and can be a T_1_-weighted magnetic resonance (MR) contrast agent. Mapping *in vivo* decay rates of CmP in an SCC tumor loaded on a mouse thigh was measured using MR imaging (MRI).^(18)^ The tumor tissue showed a faster decay rate of MR intensity compared with normal tissue and was similar to the EPR results. Good temporal MRI resolution makes dynamic imaging of TEMPOL possible.^(8,19)^ The decay rate of TEMPOL in MR intensity was similar for both normal muscle and salivary glands; however, the decay rate was significantly faster in the tumor region.^(8)^

Takeshita *et al.*^(20)^ reported that the rate constants of the nitroxyl radical reactions with ^•^OH were in the order of 10^9^ M^−1^s^−1^ and that with O_2_^•−^ in the presence of cysteine was 10^3^–10^4^ M^−1^s^−1^. It is unclear whether this high reaction rate constant between nitroxyl radicals and ^•^OH is due to the reaction specificity of nitroxyl radicals or the result of a disordered reactivity of ^•^OH. That is, a higher reaction rate constant with ^•^OH may not be due to the efficient neutralization of ^•^OH. Furthermore, each nitroxyl radical has a reactive specificity with O_2_^•−^. The normalized reduction rates of TEMPOL and CmP (0.1 mM) reacted with O_2_^•−^ generated using the hypoxanthine and xanthine oxidase (HX-XO system) (0.02 U/ml) in air-saturated phosphate buffer were 0.88 and 0.17, respectively.^(21)^ When the 6-membered ring nitroxyl radicals were compared, the reaction specificity of the nitroxyl radicals with other reductants was found to be dependent on Gibbs energy.^(22)^ However, studies regarding the competitive reaction specificity of nitroxyl radicals at specific doses of ROS are still in progress.

In this study, the amount of one-electron oxidation of TEMPOL, CmP, and their hydroxylamine forms (TEMPOL-H and CmP-H, respectively) in ^•^OH and O_2_^•−^ atmospheres were compared. To allow for comparison, the amount of spin-trapped O_2_^•−^ in the HX-XO reaction system and the amount of spin-trapped ^•^OH generated from H_2_O_2_ by UV irradiation was adjusted to be equal.

## Materials and Methods

### Chemicals

TEMPOL, CmP, xanthine oxidase (from bovine milk), and hypoxanthine were purchased from Sigma-Aldrich Co. (St. Louis, MO). GSH and H_2_O_2_ were purchased from Wako Chemical Co. (Tokyo, Japan). TEMPOL-H and CmP-H were gifts from Dr. Murali C. Krishna (Radiation Biology Branch, NCI, NIH, Bethesda, MD). A spin-trapping agent, 5-(2,2-dimethyl-1,3-propoxycyclophosphoryl)-5-methyl-1-pyrroline-*N*-oxide (CYPMPO) was a gift from Mr. Masato Kamibayashi (Kyoto Pharmaceutical University).^(23)^ The other chemicals were of analytical grade. The basic solvent for preparing reaction mixtures was 100 mM phosphate buffer (PB; pH 7.0) containing 0.05 mM diethylenetriaminepentaacetic acid (DTPA) (100 mM PB), and this was used for all experiments. Deionized water (Milli-Q system) was used to prepare 100 mM PB.

### Adjustment of the amount of ROS generation

O_2_^•−^ was generated from the reaction between hypoxanthine and xanthine oxidase (HX-XO system). An aliquot of the hypoxanthine solution was added (final concentration 0.5 mM) to initiate a reaction in a mixture containing 1 U/ml xanthine oxidase and 0.5–100 mM CYPMPO. An aliquot of the reaction mixture (120–130 µl) was sampled in a quartz flat cell. The flat cell containing the sample solution was then placed in a TE-mode cavity that was attached to an X-band EPR spectrometer (JEOL, Akishima, Tokyo), and the EPR spectrum was measured immediately. The X-band EPR signal derived from the O_2_^•−^-adduct of CYPMPO (CYPMPO-OOH) was measured repeatedly using the EPR conditions described below.

**Fig. 2:**
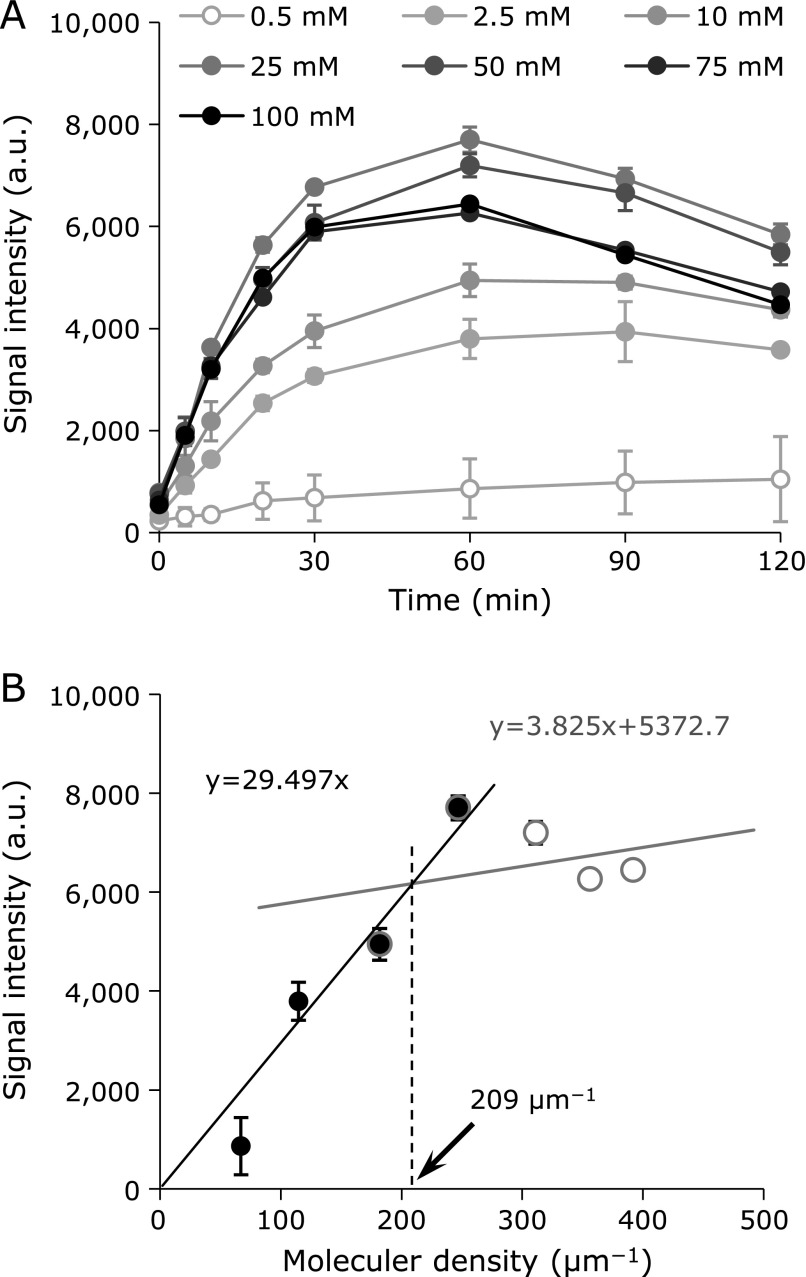
Saturation of CYPMPO-OOH detection observed when the concentration of CYPMPO in the reaction mixture was increased in a step-wise manner. (A) Time course of CYPMPO-OOH generation with several concentrations of CYPMPO. (B) Plot of the molecular density of CYPMPO in the reaction mixture versus EPR signal intensity obtained at 60 min after starting the reaction. CYPMPO-OOH generation appeared saturated when the CYPMPO density was higher than 209 µm^−1^, which is corresponding to a concentration of 15.1 mM.

**Fig. 3:**
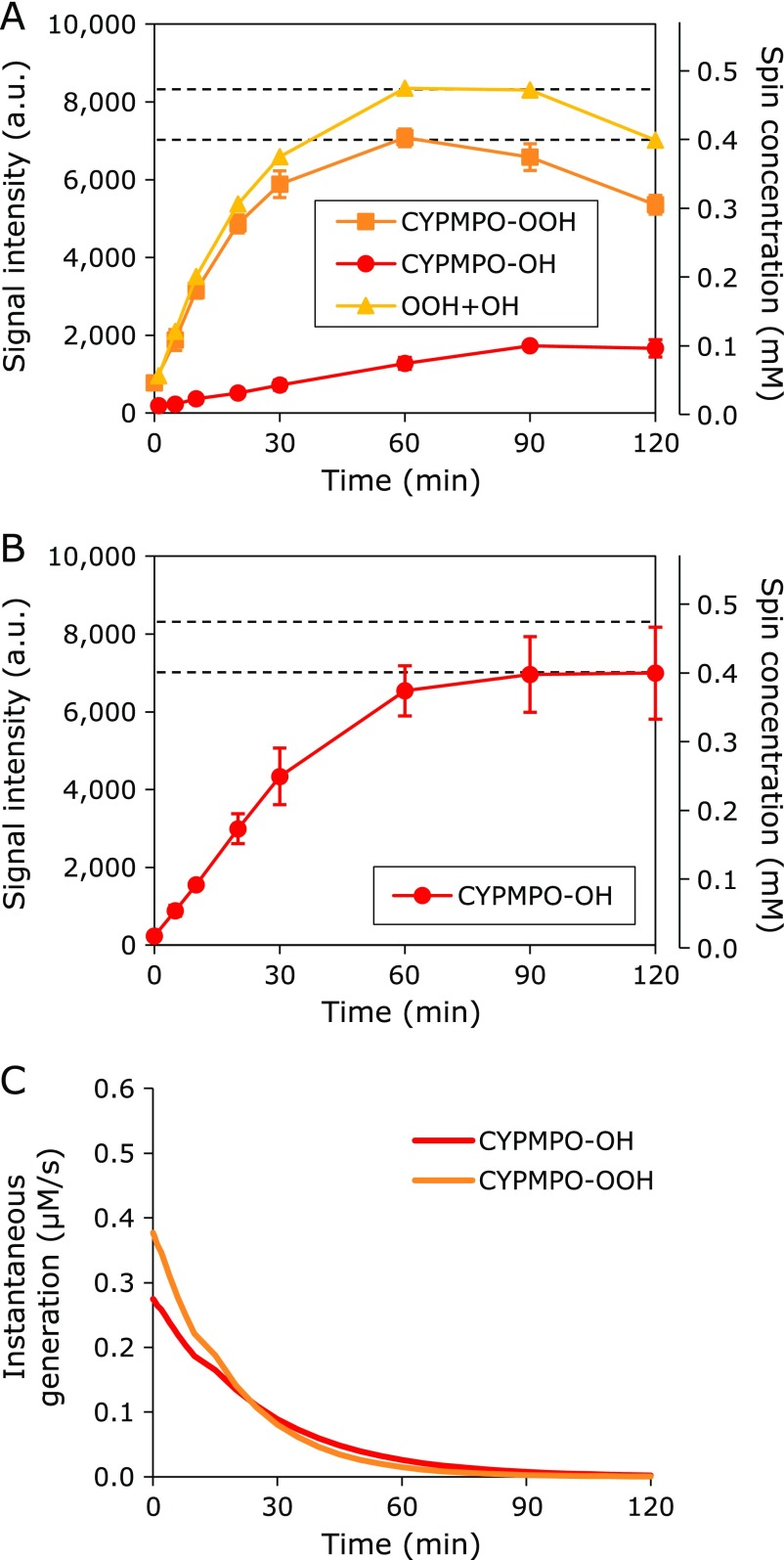
Time course comparisons between O_2_^•−^ and ^•^OH generation. (A) O_2_^•−^ was generated by reacting hypoxanthine and xanthine oxidase together in the reaction mixture (HX-XO system). (B) ^•^OH was generated from H_2_O_2_ by UV irradiation of the reaction mixture (H_2_O_2_-UV system). (C) Time course of instantaneous generation of CYPMPO-ROS adducts, i.e., differentiation of the corresponding accumulation profiles. The O_2_^•−^ and/or ^•^OH generated in the reaction mixture was spin-trapped using CYPMPO. The generation of CYPMPO-OH in the H_2_O_2_-UV system (circle) was adjusted to be similar to the generation of CYPMPO-OOH in the HX-XO system (square) at the plateau stage.

From the results shown in Fig. 2, the concentration of CYPMPO in the experiments described below was set at 50 mM, which was probably sufficient to detect most of the O_2_^•−^ generated in the HX-XO system. The experiment was repeated using 50 mM CYPMPO, and then the time courses of the EPR signal intensity derived from the O_2_^•−^-adduct of CYPMPO (CYPMPO-OOH) and the ^•^OH-adduct of CYPMPO (CYPMPO-OH) were obtained separately (Fig. 3A).

^•^OH was generated from H_2_O_2_ by UVB irradiation (H_2_O_2_-UV system). An aliquot of the reaction mixture (120–130 µl) containing 1 mM H_2_O_2_ and 50 mM CYPMPO was sampled in a quartz flat cell. The flat cell containing the sample solution was subsequently placed in a TE-mode cavity using a special cell holder. The reaction mixture was then irradiated by UVB (290 nm) using a UV irradiation unit (JEOL) equipped onto the TE-mode cavity. The time courses of the EPR signal intensity derived from the ^•^OH-adduct of CYPMPO (CYPMPO-OH) were obtained (Fig. 3B). Using the diaphragm, the UV intensity was adjusted, and the H_2_O_2_-UV experiments were repeated until the concentration of CYPMPO-OH in the H_2_O_2_-UV system (circle in Fig. 3B) was similar to the concentration of CYPMPO-OOH in the HX-XO system (square in Fig. 3A) at plateau. The UV irradiation parameters were fixed during all the experiments.

### Reactivity of the nitroxyl radical and ROS

An aliquot of a hypoxanthine solution was added (final concentration 0.5 mM) to initiate a reaction mixture containing 1 U/ml xanthine oxidase, 1 mM GSH, and 0.1 mM TEMPOL or CmP. An aliquot of the reaction mixture (120–130 µl) was sampled in a quartz flat cell. The flat cell containing sample solution was subsequently placed in a TE-mode cavity using a special cell holder, and the EPR spectrum was measured immediately. The time courses of EPR signal decay of TEMPOL or CmP were observed using the EPR conditions described below.

An aliquot of the reaction mixture (120–130 µl) containing 1 mM H_2_O_2_, 1 mM GSH, and 0.1 mM TEMPOL or CmP was sampled in a quartz flat cell. The flat cell containing the reaction mixture was then placed in a TE-mode cavity and subsequently irradiated by UV irradiation. The time courses of the EPR signal decay of TEMPOL or CmP was observed using the EPR conditions described below.

### Reactivity of hydroxylamine and ROS

An aliquot of a hypoxanthine solution was added (final concentration 0.5 mM) to initiate a reaction mixture containing 1 U/ml xanthine oxidase and 0.1 mM TEMPOL-H or CmP-H. An aliquot of the reaction mixture (120–130 µl) was sampled in a quartz flat cell. The flat cell containing the sample solution was then placed in a TE-mode cavity using a special cell holder, and the EPR spectrum was measured immediately. The time courses of the EPR signal for TEMPOL or CmP were observed using the EPR conditions described below.

An aliquot of the reaction mixture (120–130 µl) containing 1 mM H_2_O_2_ and 0.1 mM TEMPOL-H or CmP-H was sampled in a quartz flat cell. The flat cell containing the reaction mixture was placed in a TE-mode cavity and then irradiated by UV. The time courses of EPR signal for TEMPOL or CmP were observed subsequently.

### X-band EPR measurement

The X-band EPR conditions were as follows: microwave frequency, 9.4 GHz; microwave power, 4 mW; center field, 334 mT; sweep width, 10 mT; sweep speed, 5 mT/min; modulation frequency, 100 kHz; modulation amplitude, 0.079 mT; and time constant, 0.03 s.

## Results and Discussion

Figure 2A shows the accumulation profiles of CYPMPO-OOH in the HX-XO reaction system obtained using several different concentrations of CYPMPO. When the CYPMPO concentration in the reaction mixture was increased, the amount of CYPMPO-OOH detected increased with the CYPMPO concentration. The maximum EPR signal intensities of CYPMPO-OOH obtained at 60 min after starting reaction were plotted versus the molecular density of CYPMPO (Fig. 2B). The molecular density was defined as the reciprocal of the molecule-to-molecule distance in the previous paper.^(24,25)^ Detected CYPMPO-OOH increased linearly with increasing CYPMPO density, and was saturated when the CYPMPO density was more than 209 µm^−1^, which corresponded to a concentration of 15 mM. This result suggested that most O_2_^•−^ generated in this reaction mixture could be detected using CYPMPO at a concentration of more than 15 mM. Because the inflection point of the plot was obtained at 209 µm^−1^, localized generation of O_2_^•−^ at the corresponding concentration of 15 mM was expected. The molecular density of 209 µm^−1^, i.e., concentration of 15 mM, corresponds to a molecule-to-molecule distance of 5 nm. This was the roughly the same order as the size of a subunit of xanthine oxidase reported previously, 10 × 9 × 7 nm.^(26)^ Therefore, the generation of O_2_^•−^ may be localized on the xanthine oxidase enzyme. In addition, the concentration of 50 mM used in later experiments was enough to detect most of the O_2_^•−^ generated.

Figure 3A shows the accumulation profile of CYPMPO-OOH in the HX-XO reaction system. The simultaneous accumulation of CYPMPO-OH with CYPMPO-OOH was also observed in this system. The concentration of CYPMPO-OOH increased gradually with time and reached a concentration of 0.4 mM after 60 min from the start of the reaction before gradually decaying. Similarly, the concentration of CYPMPO-OH increased gradually until 90 min after the start of the reaction and reached a plateau at 0.1 mM. The accumulation rates for CYPMPO-OH and CYPMPO-OOH in the HX-XO reaction system were 0.0207 min^−1^ and 0.0562 min^−1^, respectively.

Figure 3B shows the accumulation profile of CYPMPO-OH in the H_2_O_2_-UV reaction system. The concentration at which CYPMPO-OH plateaued was positively correlated with the level of UV intensity (data not shown). Using the diaphragm of the UV irradiation unit, the plateau level of CYPMPO-OH accumulation was adjusted to approximately 0.4 mM. The accumulation rate of CYPMPO-OH in the H_2_O_2_-UV reaction system was 0.0409 min^−1^. This UV condition was used for all subsequent experiments.

The instantaneous generation of CYPMPO-OOH and CYPMPO-OH in the very early moment of the reaction can be calculated from generation rates of 0.377 and 0.275 µM/s, respectively. Therefore, 37% more O_2_^•−^ compared with ^•^OH can be reacted with CYPMPO in the early stage of the reactions. The time course of instantaneous generation of CYPMPO-ROS adducts, i.e., differentiation of the accumulation profile, decreased gradually with time, and the lead switched after 20 min (Fig. 3C).

The accumulation rates of CYPMPO-OOH and CYPMPO-OH in the reaction system should be proportional to the generation rates of O_2_^•−^ and ^•^OH; however, this did not exactly mean the true generation rates of O_2_^•−^ and ^•^OH. Such reactive species, especially ^•^OH, can react with other molecules found in the reaction mixture and part of them could be canceled before they reached CYPMPO. The generations and reactions of O_2_^•−^ and ^•^OH with CYPMPO can be indicated as Eq. 1–4.

$$\text{HX-XO}\overset{{\;k}_{\mathrm{s1}\;}}{\to }{O_{2}}^{∙-}\overset{{\;}_{}}{\to }\text{cancelled}$$ [1]

$${O_{2}}^{∙-}+\text{CYPMPO}\overset{{\;k}_{\mathrm{s2}\;}}{\to }\text{CYPMPO-OOH}\overset{{\;k}_{\mathrm{s3}\;}}{\to }\text{degraded}$$ [2]

H2O2-UV→     kh1     OH∙→                cancelled [3]

OH∙+CYPMPO→     kh2     CYPMPO-OH→     kh3     degraded [4]

Where, *k*_s1_ and *k*_h1_ are the generation rates of O_2_^•−^ and ^•^OH, but these are difficult to estimate in this experiment; *k*_s2_ and *k*_h2_ are the generation and *k*_s3_ and *k*_h3_ are the degradation rates of CYPMPO-OOH and CYPMPO-OH, respectively.

The accumulation rate of CYPMPO-OH and CYPMPO-OOH can be indicated as shown below.

$$V_{s}=\frac{d}{dt}\left[\text{CYPMPO-OOH}]=k_{\mathrm{s2}}[{O_{2}}^{∙-}][\text{CYPMPO}]-k_{s3}[\text{CYPMPO-OOH}\right]$$ [5]

Vh=ddt[CYPMPO-OH]=kh2[OH∙][CYPMPO]-kh3[CYPMPO-OH] [6]

The half-lives of CYPMPO-OOH and CYPMPO-OH, were reported as 51 min and more than 60 min, respectively.^(23)^ The decay constants *k*_s3_ and *k*_h3_ can be calculated as 0.23 × 10^−3^ and 0.19 × 10^−3^ s^−1^ from the half-lifes, when the half-life of CYPMPO-OH was as 60 min for simplification. The decay of CYPMPO-OOH and CYPMPO-OH can be negligible, since the decay rates *k*_s3_ and *k*_h3_ are sufficiently slow. If the accumulation rates of CYPMPO-OOH and CYPMPO-OH were ascribed as being the same, *k*_s2_[O_2_^•−^] must be equal to *k*_h2_[^•^OH]. The second-order rate constants for trapping O_2_^•−^ and ^•^OH by CYPMPO were reported as 48 M^−1^s^−1^ and >10^9^ M^−1^s^−1^, respectively.^(23)^ When the trapping rate of ^•^OH was 1 × 10^9^ M^−1^s^−1^ for simplification,

ks2O2∙-=kh2OH∙∶48 M-1s-1 O2∙-=109 M-1s-1 [OH∙]. [7]

Then, [O_2_^•−^] should be roughly 20,000,000 times more than [^•^OH]. This fact suggests that only 0.000005% or less of total ^•^OH could react with CYPMPO in each moment in this reaction system. In a practical sense, however, the actual concentration difference between [O_2_^•−^] and [^•^OH] might be much smaller, since the rate-controlling factors for accumulating CYPMPO-OOH and CYPMPO-OH in the sequential reactions are definitely *k*_s1_ and *k*_h1_, respectively. It was due to that the trapping rates *k*_s2_ and *k*_h2_ were predominantly faster than the generation rates *k*_s1_ and *k*_h1_.

In 50 mM CYPMPO, the molecular distance between CYPMPO molecules was calculated as 3.2 nm, which is a longer distance than ^•^OH can travel in water. There can be 10 or more water molecules present within a 3.2 nm linear space. ^•^OH was generated from 1 mM H_2_O_2_ in the H_2_O_2_-UV reaction system in our study. The concentration of 1 mM H_2_O_2_ resulted in a molecular distance of 11.8 nm. The molecular distance between CYPMPO molecules, the detector of ^•^OH, was sufficiently shorter than the distance of ^•^OH generated, whereas a concentration of 50 mM CYPMPO was not sufficient for detecting all ^•^OH molecules generated.

This level, however, would probably be sufficient for detecting most of the O_2_^•−^ generated in the reaction mixture, as described above. The O_2_^•−^ generated in the HX-XO reaction system was derived from dissolving oxygen in the reaction mixture. The maximum possible oxygen concentration in air-saturated water at 25°C is 0.26 mM, resulting in an average molecular distance of 18.7 nm between oxygen molecules. However, O_2_^•−^ generation may be localized on the enzyme and it may corresponding to 15 mM at a local concentration.

The amount of total adduct formation (0.4 mM), which corresponded to the amount of ROS able to reach the target, was almost the same between the HX-XO and H_2_O_2_-UV systems used in this study (Fig. 3A and B). The spin-adduct formation at each instantaneous moment in the O_2_^•−^ atmosphere in the HX-XO system and ^•^OH atmosphere in the H_2_O_2_-UV system used in this paper were approximately the same (Fig. 3C). Available oxidation reactions at each instantaneous moment may be similar between these reaction systems. The use of 50 mM CYPMPO as the target molecules for oxidation is probably suitable when a congested intracellular environment is considered.^(27)^ Therefore, this experiment was actually adjusting the total oxidation reaction by ROS to target molecules in the HX-XO and H_2_O_2_-UV systems.

CYPMPO could provide more stable adducts of ROS compared with DMPO. DMPO adducts of O_2_^•−^ (DMPO-OOH) have a relatively short half-life (≈ 1 min), whereas the CYPMPO-OOH adduct has a half-life of 51 min in the HX-XO system.^(23)^ CYPMPO-OH is more stable and has a longer half-life (>60 min).^(23)^ These are sufficiently long half-lives to follow the reactions caused gradually over 60–120 min.

**Fig. 4:**
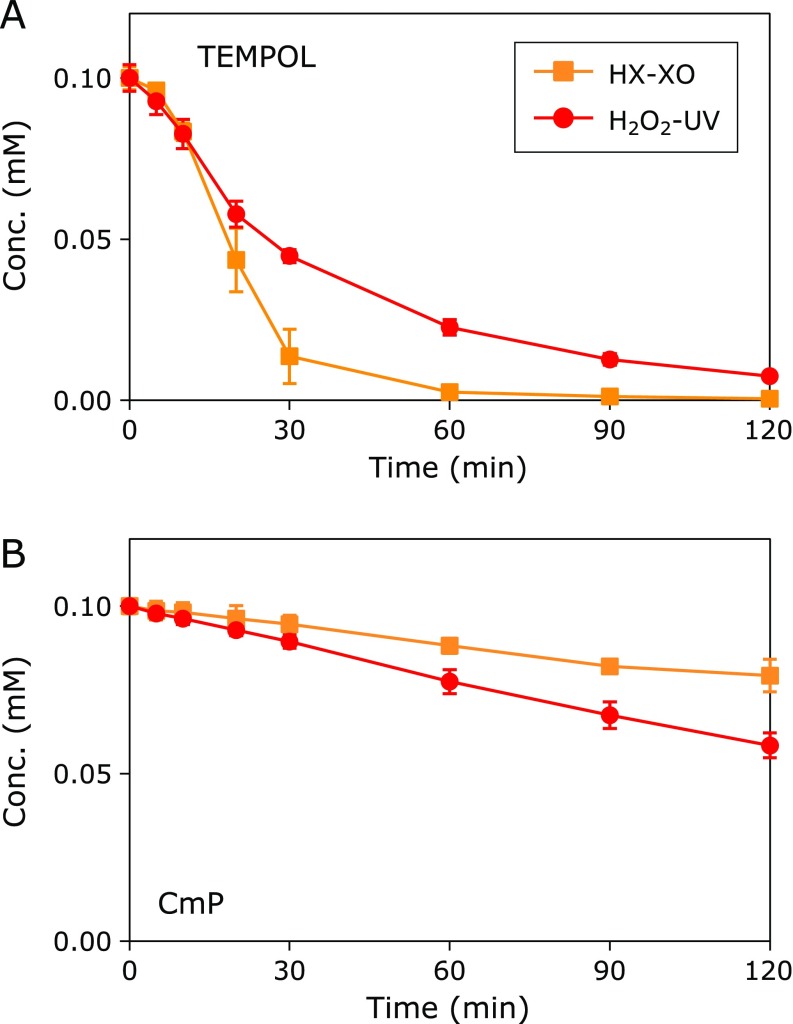
ROS-induced reduction of TEMPOL and CmP in the presence of GSH. (A) Reaction profiles of TEMPOL. (B) Reaction profiles of CmP. Squares indicate the reaction profiles in the HX-XO system. Circles indicate the reaction profile in the H_2_O_2_-UV system. Marks and error bars indicate the average ± SD of 3 experiments.

When TEMPOL or CmP was exposed to a reaction with ROS generation, the EPR signal intensity of TEMPOL or CmP decreased in the presence of GSH (Fig. 4). This reduction reaction of nitroxyl radicals was not observed in the absence of GSH (data not shown). Therefore, as shown in Fig. 1, the nitroxyl radicals were one-electron oxidized to form an oxoammonium cation and subsequently two-electron reduced to form hydroxylamine or another stable molecule. The one-electron oxidation of nitroxyl radicals in the aqueous environment of the HX-XO reaction system is most likely due to HO_2_^•^, an alternate form of O_2_^•−^. However, its population is small compared with that of O_2_^•−^.

There was a greater reduction in TEMPOL in the O_2_^•−^ atmosphere (HX-XO reaction system) than in the ^•^OH atmosphere (H_2_O_2_-UV reaction system) (Fig. 4A). In other words, TEMPOL is more oxidized in environments containing HO_2_^•^ than those with ^•^OH. When the ROS generation was adjusted to reach a plateau at approximately 0.4 mM after 60 min, approximately 0.1 mM TEMPOL could be reduced during this time in the HX-XO reaction system, whereas 0.08 mM TEMPOL could be reduced in the H_2_O_2_-UV system. A greater amount of CmP was reduced in the ^•^OH atmosphere (H_2_O_2_-UV reaction system) than in the O_2_^•−^ atmosphere (HX-XO reaction system) (Fig. 4B). Therefore, CmP is oxidized to a greater extent in a reaction with ^•^OH than with HO_2_^•^. Considering the population of HO_2_^•^, the nitroxyl radicals, especially TEMPOL, preferably react with HO_2_^•^.

In general, CmP is more redox stable compared with TEMPOL. The redox potentials of TEMPOL and CmP reported by Krishna *et al.*^(21)^ and Kinoshita *et al.*^(22)^ suggested that CmP is more resistant against reduction. Another reason for the stability of CmP is the stearic hindrance of the nitroxyl radical moiety. The 5-membered ring structure of CmP is fixed and kept in a plane ring, whereas the 6-membered ring of TEMPOL can bend and exchange between boat and chair forms. The free radical on the pie-orbital of the N-O bond of CmP was always hidden by 4 methyl moieties on neighbor carbons, whereas the free radicals on TEMPOL can peep from the hindrance by methyl moieties due to the bending of the ring formation. Therefore, the free radical on TEMPOL has more chance for a reaction. However, the exact mechanism of the faster reaction of TEMPOL with HX-XO is unclear.

**Fig. 5:**
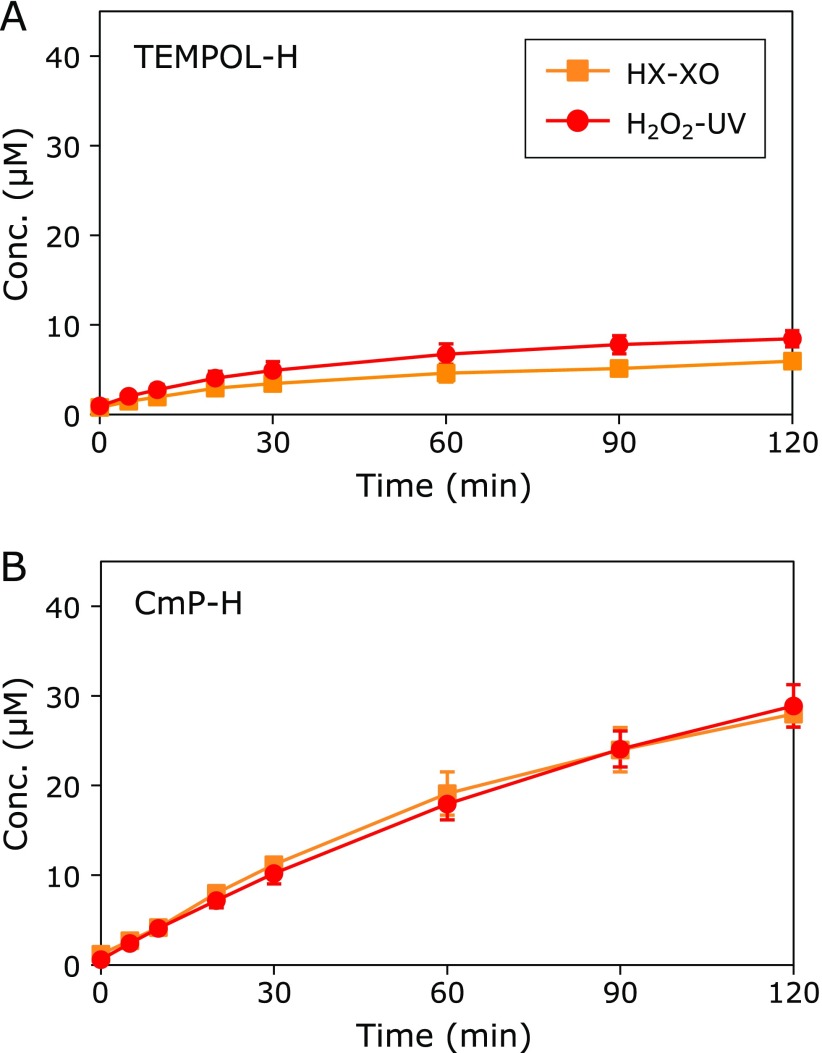
ROS induced oxidation of TEMPOL-H and CmP-H. (A) Reaction profiles of TEMPOL-H. (B) Reaction profiles of CmP-H. The squares indicate the reaction profiles in the HX-XO system. Circles indicate the reaction profile in the H_2_O_2_-UV system. Marks and error bars indicate the average ± SD of 3 experiments.

Figure 5 shows the reactivity between hydroxylamines and ROS. The one-electron oxidation of hydroxylamines to the corresponding nitroxyl radical occurs almost evenly in both the ^•^OH (H_2_O_2_-UV reaction system) and O_2_^•−^ atmospheres (HX-XO reaction system). Reactions between TEMPOL-H and ROS occurred at a rate of less than 1/10 of those that occurred between TEMPOL and ROS. There was no difference in the oxidation reactivity of CmP-H in the ^•^OH or O_2_^•−^ atmospheres. The amount of oxidized CmP-H was approximately 3 times greater than that of TEMPOL-H.

TEMPOL could be oxidized to the corresponding oxoammonium cation form relatively quickly, and then sequentially reduced to a diamagnetic species with coexisting GSH. However, the oxidation of TEMPOL-H, which is a one-electron reduced form of TEMPOL, was substantially slower, when similar amounts of oxidizing ROS were provided. Moreover, TEMPOL could be more sensitively oxidized in the O_2_^•−^/HO_2_^•^ atmosphere compared with the ^•^OH atmosphere. Therefore, the accelerated reduction of TEMPOL *in vivo* may be a good candidate as a redox probe for O_2_^•−^-induced oxidative stress. Because O_2_^•−^-induced oxidative stress may be caused by an error during electron transfer in mitochondrial energy production, the amphiphilicity of TEMPOL is convenient as a probe in such intracellular patho-physiological conditions. On the other hand, CmP as an *in vivo* redox probe is probably sensitive enough to show the existence or absence of the reoxidation process of CmP-H to CmP, which may be caused in extracellular conditions. Investigators should choose a suitable nitroxyl redox probe for the purpose of the investigation according to its characteristic redox behavior.

## Conclusion

By generating similar amounts of ^•^OH or O_2_^•−^ in reaction mixtures containing different types of nitroxyl radicals, the reaction specificity of nitroxyl radicals for a particular ROS can be estimated. TEMPOL was more easily oxidized in an O_2_^•−^ atmosphere than in an ^•^OH atmosphere, whereas the opposite was observed for CmP. Although the hydroxylamine forms of TEMPOL-H and CmP-H were oxidized evenly in the ^•^OH or O_2_^•−^ atmospheres, the amount of oxidized CmP-H was 3 times greater than that of TEMPOL-H.
